# Supplementary figures and images for: Characteristic Dissection of Xanthomonas oryzae pv. oryzae Responsive MicroRNAs in Rice
Source: Int J Mol Sci. 2020 Jan 25;21(3):785. doi: 10.3390/ijms21030785 (PMC7037501; doi:10.3390/ijms21030785)

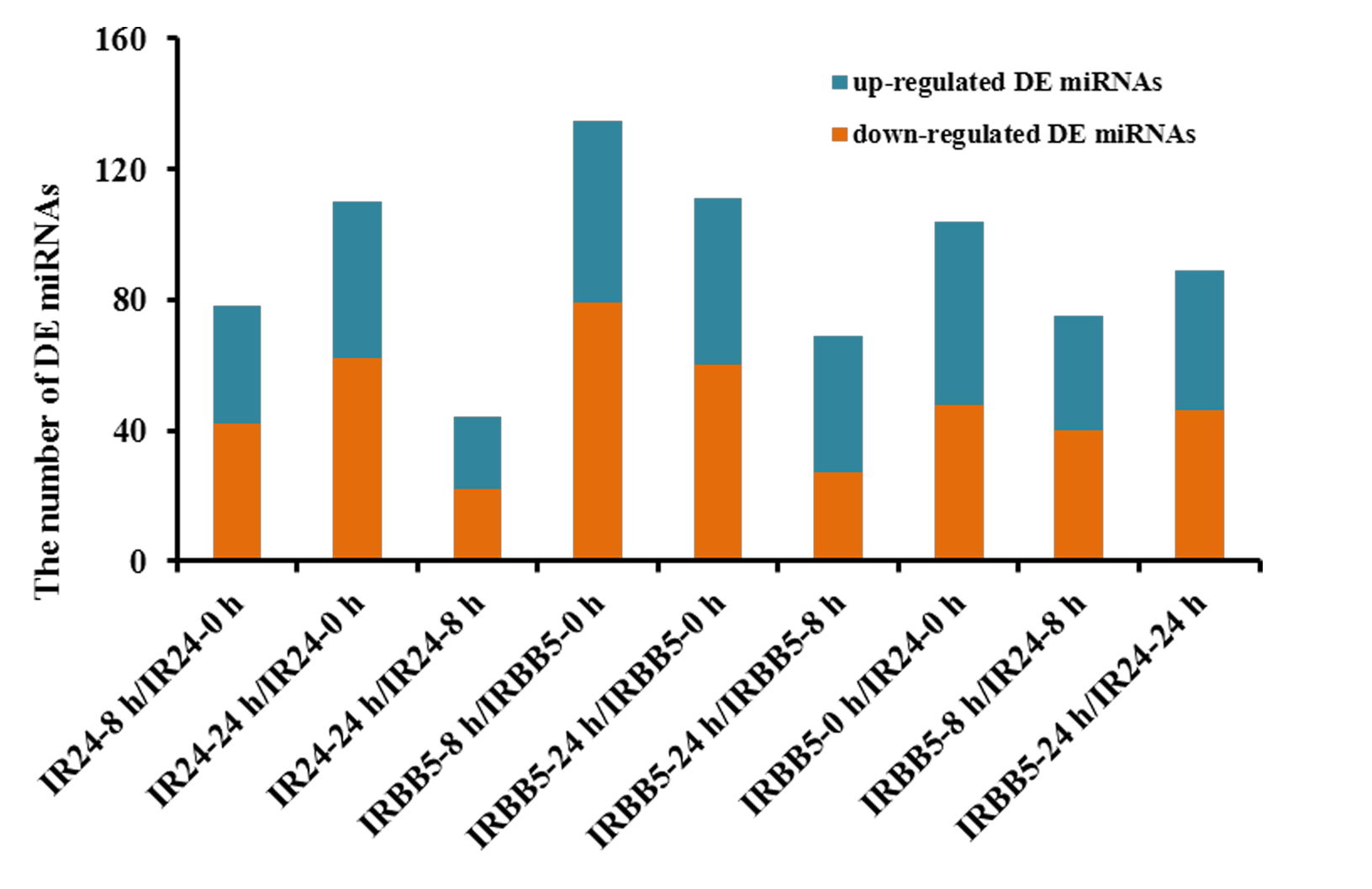

Supplement: Supplementary file 1 [file ijms-21-00785-s001.zip › supplementary files/Figure S1.tif]

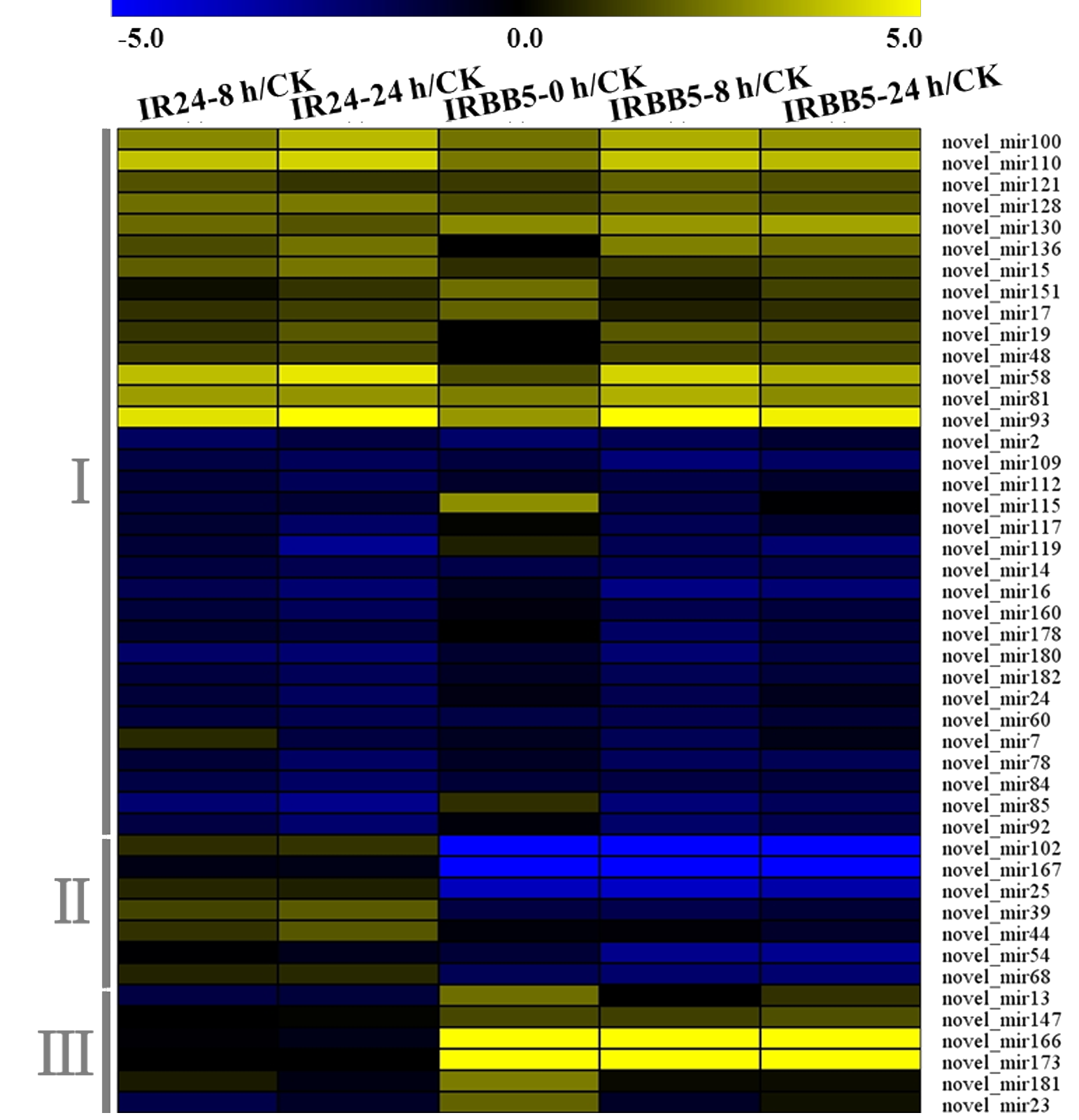

Supplement: Supplementary file 1 [file ijms-21-00785-s001.zip › supplementary files/Figure S2.tif]

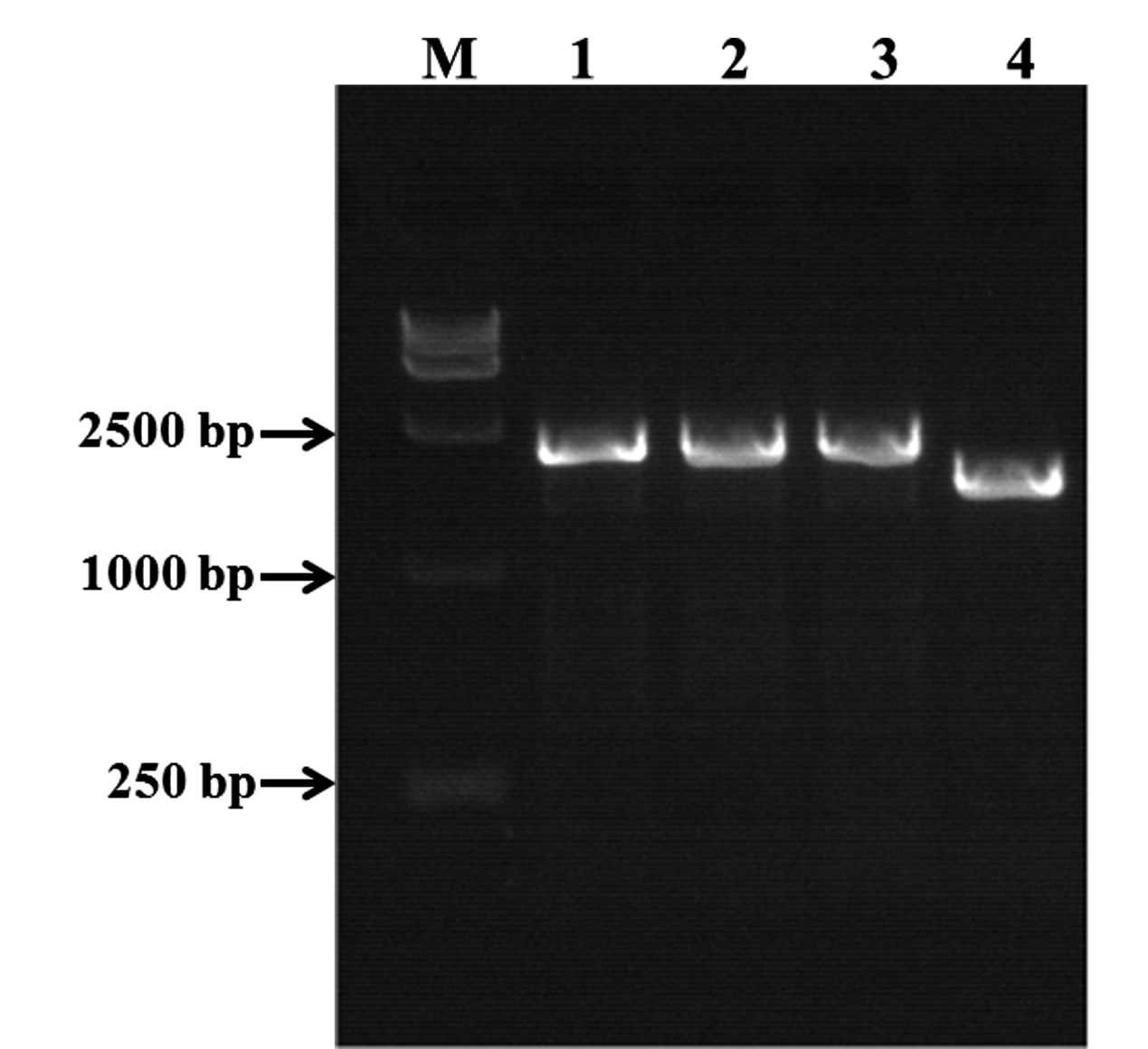

Supplement: Supplementary file 1 [file ijms-21-00785-s001.zip › supplementary files/Figure S3.tif]
